# Diamond Structures for Tuning of the Finesse Coefficient of Photonic Devices

**DOI:** 10.3390/ma15072552

**Published:** 2022-03-31

**Authors:** Monika Kosowska, Awadesh K. Mallik, Michał Rycewicz, Ken Haenen, Małgorzata Szczerska

**Affiliations:** 1 Faculty of Telecommunications, Computer Science and Electrical Engineering, Bydgoszcz University of Science and Technology, Al. Prof. S. Kaliskiego 7, 85-796 Bydgoszcz, Poland; 2 Solvay Business School, Vrije Universiteit Brussel, Pleinlaan 2, 1050 Brussels, Belgium; awadesh.kumar.mallik@vub.be; 3 Department of Metrology and Optoelectronics, Faculty of Electronics, Telecommunications and Informatics, Gdańsk University of Technology, 11/12 Narutowicza Street, 80-233 Gdansk, Poland; micrycew@student.pg.edu.pl; 4 Institute for Materials Research (IMO), Hasselt University and IMOMEC, IMEC vzw, Wetenschapspark 1, 3590 Diepenbeek, Belgium; ken.haenen@uhasselt.be

**Keywords:** diamond, CVD, cavity, optoelectronic devices, fiber-optic sensor

## Abstract

Finesse coefficient is one of the most important parameters describing the properties of a resonant cavity. In this research, a mathematical investigation of the application of diamond structures in a fiber-optic Fabry–Perot measurement head to assess their impact on the finesse coefficient is proposed. We present modeled transmission functions of cavities utilizing a nitrogen-doped diamond, a boron-doped diamond, nanocrystalline diamond sheet and a silver mirror. The diamond structures were deposited using a microwave plasma-assisted chemical vapor deposition system. A SEM investigation of surface morphology was conducted. The modeling took into consideration the fiber-optic Fabry–Perot setup working in a reflective mode, with an external cavity and a light source of 1550 nm. A comparison of the mathematical investigation and experimental results is presented.

## 1. Introduction

The growing interest in optical measurements is related to many benefits this approach offers. Optical techniques are non-contact and do not damage the investigated samples due to their non-destructive working manner. This can be crucial for measurements where direct contact of the probe may alter or damage the sample. Optical methods assure fast operation and allow a real-time monitoring. Moreover, they exhibit a great potential for coupling with other methods, giving multi-mode systems capable of providing more comprehensive results [1,2].

Among numerous sensing solutions, interferometric fiber-optic sensors are of greatest interest as they provide high sensitivity, resolution and dynamic range of measurements [3]. They are immune to electromagnetic and radio frequency interference as well as being spark-free because the measurement head only uses light [4]. Small weight and dimensions allow their installation in challenging locations and environmental conditions. Depending on the selected configuration they can serve as pointwise or distributed sensors with ease to couple with existing telecommunication systems [5]. While applying a broadband light source or a wavelength-tunable laser, we achieve absolute values of the measurand [6].

A Fabry–Perot fiber-optic interferometer offers relatively simple and cheap configuration. Its cavity is created between two partially reflective parallel mirrors with a gap between them: such configuration forms a multi-beam interferometer. However, it can be simplified and approximated to a two-beam interferometer by tailoring its cavity. Application of mirrors with low reflectivity for the cavity construction leads to a low-finesse interferometer that can be considered as a two-beam interferometer, because higher-order reflections do not significantly contribute to the resulting spectrum, and hence can be neglected [7]. The reasoning behind such interferometers lies in their robustness, simplified fabrication process and cost reduction, while maintaining high measurement resolution and sensitivity. Therefore, low-finesse Fabry–Perot interferometers successfully serve as sensors [8,9,10,11].

Finesse is the ratio of the phase separation of neighboring maxima (called the free spectral range—FSR) and the full width at half maximum—FWHM [12]. Effectively, it indicates how many times the beam bounces inside the cavity before it gets transmitted out, absorbed or scattered [13]. The finesse can be tailored depending on our needs: low finesse cavities can be applied for filtering, while high finesse cavities can serve for precise spectroscopy [14,15]. There are several factors that impact the finesse value: reflectance of the mirrors, microroughness of their surfaces, coating non-uniformities, scattering (defects after polishing or dust particles) and losses (non-parallelism between the surface, divergence of the incident light). Lots of these shortcomings were compensated by the use of new constructions of mirrors (e.g., curved, spherical ones [16,17]).

Nowadays, researchers investigate possibilities of tuning properties of the measurement devices by applying new materials for their construction. The synergy between optoelectronics and material engineering leads to solutions tailored for specific applications [18]. A great amount of attention is paid to diamond structures due to their unique properties [19], making them willingly used in the construction of sensors and elements of measuring heads [20,21,22,23]. Our previous research [24] presented the viability of diamond application in Fabry–Perot interferometers as reflective surfaces with increased immunity to mechanical and chemical damage, biocompatibility and prolonged lifespan. As a CVD-deposited-doped diamond also has satisfactory optical and electrochemical properties, it was possible to develop an opto-electrochemical setup where boron-doped diamond played a dual role of a reflective surface and a working electrode. The electrochemical solutions under test can have different optical properties and therefore the visibility of the registered optical spectra may be not sufficient. Hence, there is a need for tailoring of the finesse coefficient of the Fabry–Perot cavity with diamond structures in order to adjust it to investigated chemical solutions [25]. In this research, we mathematically investigate the application of diamond structures in Fabry–Perot cavities to tune their finesse. The optimalization of optical properties of the cavity with diamond structures in the considered setup will allow us to achieve a desired contrast of the interferometric fringes of the investigated liquids.

In this study, a transmission function of cavities utilizing a nitrogen-doped diamond, a boron-doped diamond and a silver mirror are presented. The simple and fast procedure of tailoring the finesse by exchanging diamond structures is an advantage of our setup: the properties of the structures can be tailored to desired needs by adjusting the CVD parameters process, e.g., by changing deposition time or dopant level. The proposed cavity is also compact and robust with the possibility of changing its length from 0 to 1 mm.

## 2. Mathematical Investigation

We consider a fiber-optic Fabry–Perot interferometer working in a reflective mode, with an external air-gap cavity. Such construction allows an easy access for liquid sample injection and full configurability in changing the mirrors. The interferometer is constructed of two parallel, partially reflecting surfaces M_1_ and M_2_, separated from each other with a small gap. Those two plane mirrors with reflectance R_1_ and R_2_ and the separation of length L create a cavity that traps the light at specific resonance frequencies in the form of a standing wave [13]. The light entering the cavity through the first surface R_1_ is partially reflected and partially transmitted through it. The transmitted light, propagating inside the cavity, is then partially reflected from the surface R_2_ and partially transmitted. The reflected light undergoes further partial reflections and transmissions.

The first reflective surface M_1_ is created on the interface fiber end-face/medium inside cavity (here air, n = 1) and the second surface M_2_ on the boundary between the medium and tested mirror. Since the reflectivity of the investigated samples is low and the cavity length is greater than the operating wavelength, a two-beam interferometer was assumed [26].

The reflectivity R of the surfaces creating the cavity is dependent on the refractive indices n_1_ and n_2_. As the properties of diamond structures can be tailored to desired needs by adjusting CVD process parameters (e.g., by changing dopant element or dopant level which results in changes of refractive index of the diamond sample), we can change the mirror reflectivity [27]:

(1) $$R={\left(\frac{n_{1}-n_{2}}{n_{1}+n_{2}}\right)}^{2}$$

The finesse coefficient F for an interferometer built with two asymmetrical mirrors characterized by R_1_ and R_2_ can be described as [27]:

(2) $$F=\frac{4\sqrt{R_{1}R_{2}}}{{\left(1-\sqrt{R_{1}R_{2}}\right)}^{2}}$$

where R_1_ and R_2_ describe reflectivities of the mirrors. The finesse coefficient F is therefore a function of reflectivity. With reflectivity closer to the unity, the finesse coefficient becomes bigger and, in consequence, the minima of the transmitted light characteristics decrease, resulting in narrower peaks. The sharpness of the obtained fringes can be described by their full width at half maximum (FWHM). The ratio of the phase separation of neighboring fringes—free spectral range—and the FWHM is called the finesse. The value of FSR and FWHM ratio (i.e., the finesse) depends on the reflectivities of the reflective surfaces used for the construction of the cavity [28].

## 3. Results

**Figure 1:**
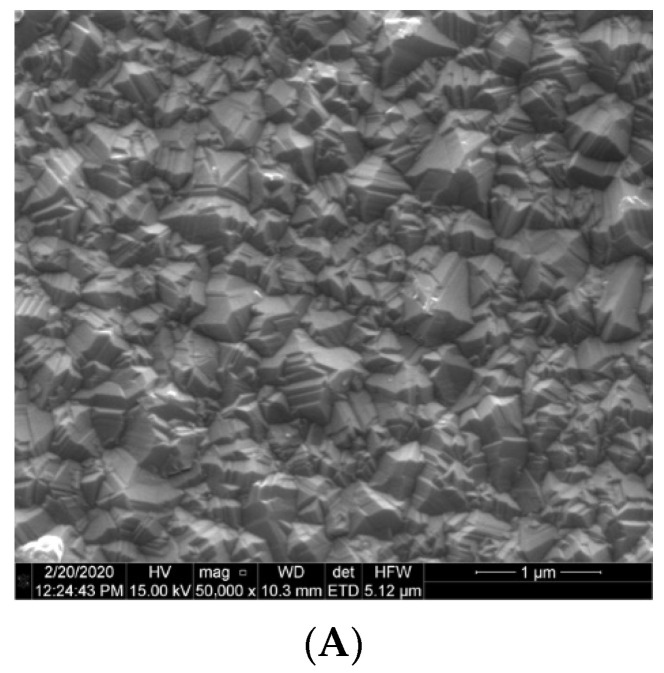

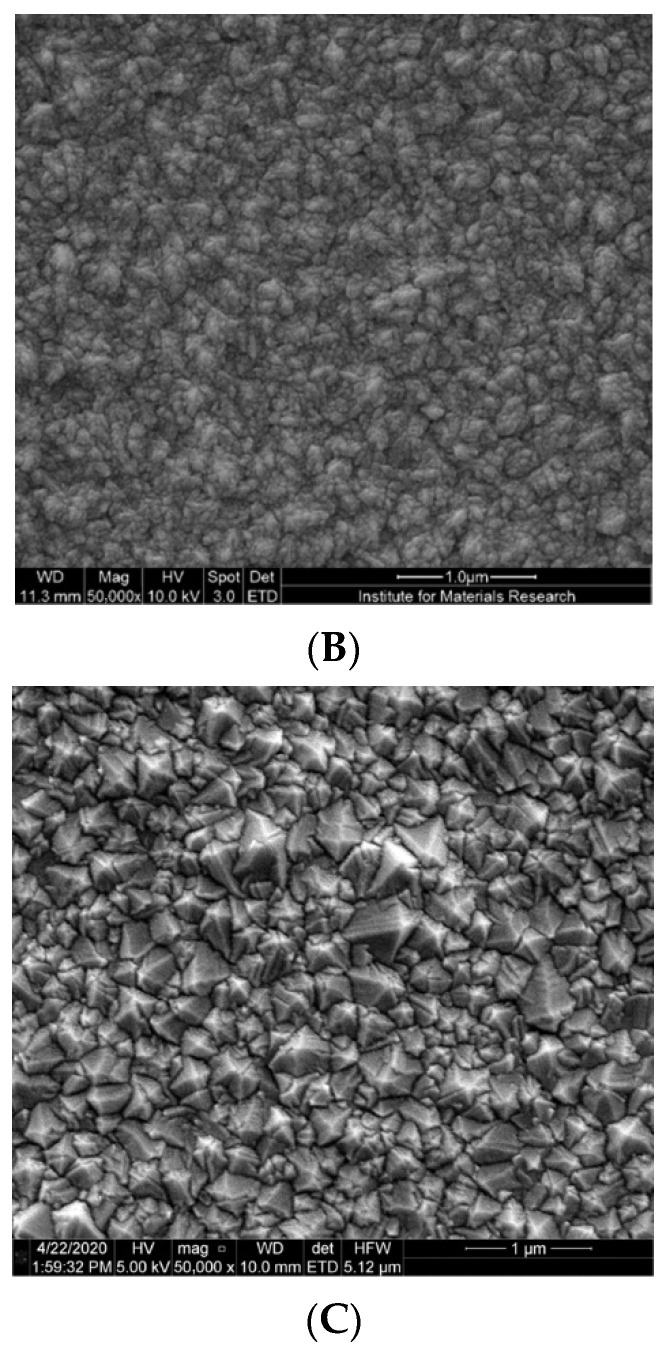
SEM images of (**A**) boron-doped diamond film, (**B**) nitrogen-doped diamond film and (**C**) nanocrystalline diamond sheet.

The investigation is based on diamond structures produced during a microwave plasma-assisted chemical vapor deposition process. The details about the growth parameters and chemicals used, as well as the investigation of the material properties can be found elsewhere [29,30,31]. The representative SEM images characterizing the structures’ surfaces are presented in Figure 1.

It is worth noting that all structures have crystalline character with uniform crystallites size and distribution over the silica substrate. The diamond grows uniformly on the substrates, covering them entirely which is crucial for the application in a Fabry–Perot cavity.

The cavities built with mirrors with highly reflecting mirrors assure high values of the finesse resulting in narrower transmittance peaks in comparison to mirrors with lower reflectivities. The intensity of the reflected light I_r_ is expressed by [27,28]:

(3) $$I_{r}=\frac{\left(2-2\cos\delta \right)R}{1+R^{2}-2\mathrm{Rcos}\delta }I_{i}=\frac{4{\mathrm{Rsin}}^{2}\frac{\delta }{2}}{{\left(1-R\right)}^{2}+4{\mathrm{Rsin}}^{2}\frac{\delta }{2}}I_{i}$$

where R is the reflectivity, δ is the phase difference and I_i_ is the incident light intensity.

The corresponding intensity of the transmitted light I_t_ is:

(4) $$I_{t}=\frac{T^{2}}{1+R^{2}-2\mathrm{Rcos}\delta }I_{i}=\frac{T^{2}}{{\left(1-R\right)}^{2}+4{\mathrm{Rsin}}^{2}\frac{\delta }{2}}I_{i}=\frac{{\left(1-R\right)}^{2}}{{\left(1-R\right)}^{2}+4{\mathrm{Rsin}}^{2}\frac{\delta }{2}}I_{i}$$

where R is the reflectivity, T is the transmission, δ is the phase difference between interfering beams and I_i_ is the incident light intensity.

In this study, we focused on the configuration of the Fabry–Perot cavity where fiber-optic end-face is used as one of the cavity interfaces. For this reason, only perpendicular light incidence is considered: the first interface is created by the polished fiber-optic end-face/medium inside the cavity, and the second is medium inside the cavity/diamond structure for configurations with diamond films. As the reflective surfaces have to be parallel in such construction, the slanted angle of light incidence should not occur as the fiber is placed in a micromechanical setup for proper positioning and stabilization. Using the aforementioned formulas, we can model the transmission of the Fabry–Perot cavity with regard to different values of the finesse coefficients.

**Figure 2:**
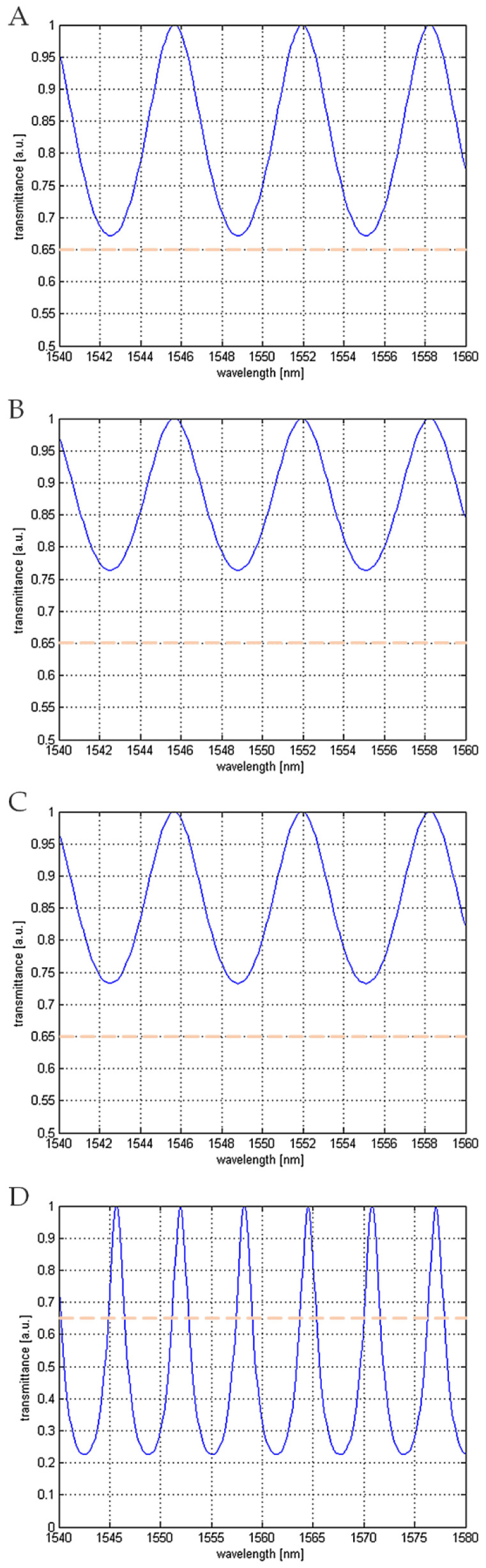
Normalized transmittance characteristics. (**A**) Silver mirror, (**B**) boron-doped diamond film, (**C**) nitrogen-doped diamond film and (**D**) nanocrystalline diamond sheet with silver mirror. Orange lines are meant to guide an eye.

The following plots (Figure 2) show results of theoretical modeling of the cavities built with a fiber end-face and the investigated mirror, with the air fulfilling the gap between them.

The parameters of the obtained plots are presented in Table 1.

**Figure 3:**
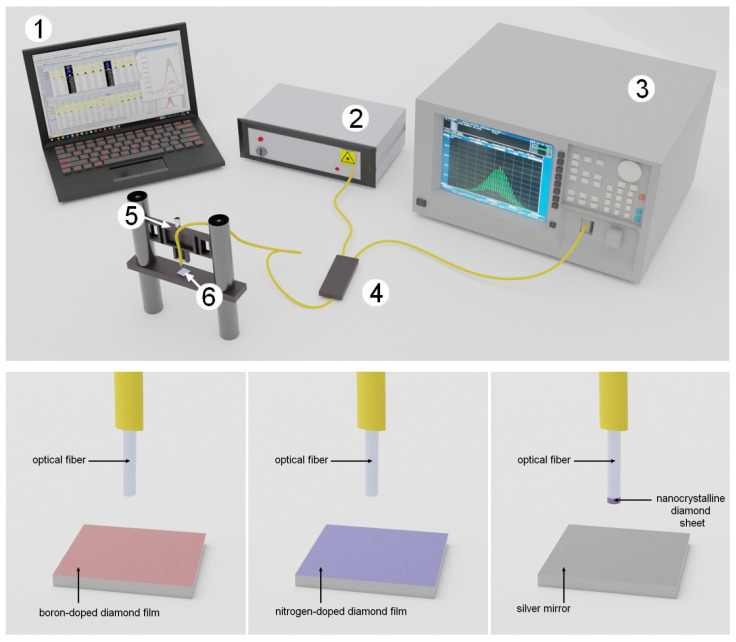
Measurement setup for the cavity parameters assessment. 1—PC; 2—light source; 3—optical spectrum analyzer; 4—2 × 1 fiber coupler; 5—micromechanical setup; 6—cavity; R_1_–R_6_—surfaces reflectivities.

To assess the quality of the models, we directly compare the results of the measurements taken with the Fabry–Perot interferometer applying the investigated samples. The setup and procedure of the measurement were described in detail elsewhere [32]. The broadband light source that was used, while performing experiments, operated at the central wavelength of 1550 nm. The scheme of the measurement setup and three main configurations are presented in Figure 3.

**Figure 4:**
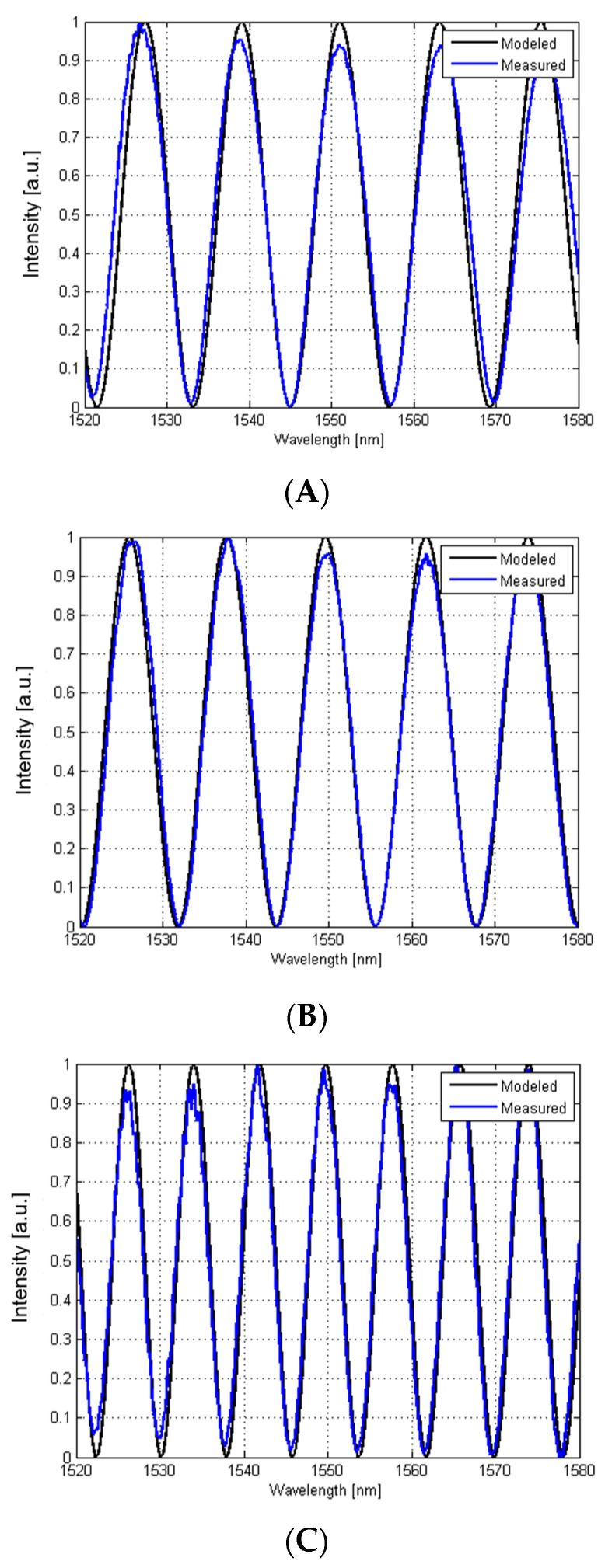

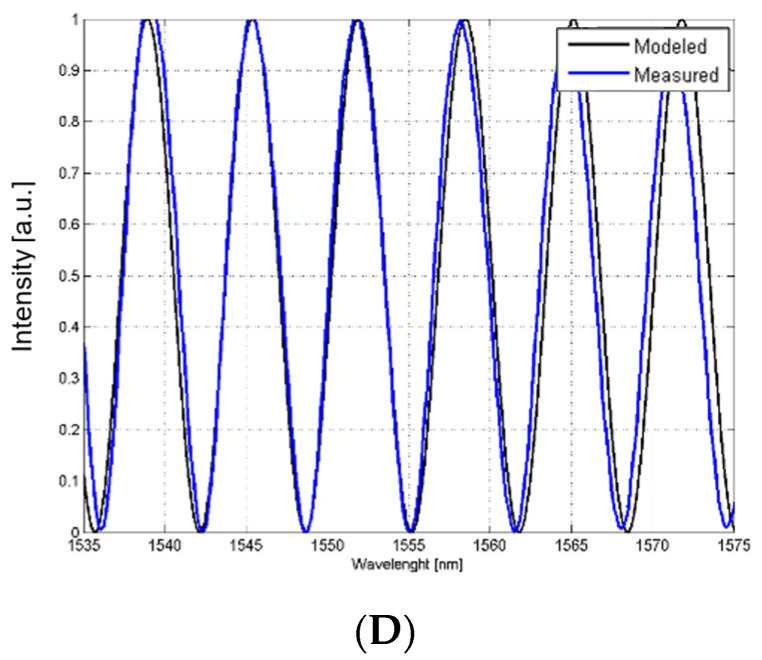
Comparison between the modeled and the measured spectra after removing the Gaussian characteristics and normalization. The cavities were fulfilled with air (n = 1). (**A**) silver mirror d = 100 µm, (**B**) boron-doped diamond film d = 100 µm, (**C**) nitrogen-doped diamond film d = 150 µm and (**D**) nanocrystalline diamond sheet with silver mirror d = 180 µm.

The Fabry–Perot cavities were filled with air (n = 1) in each case. For each spectrum, the Gaussian light source characteristics were filtered out. The modeled and the measured signals A–D (Figure 4) were achieved for the following settings (Table 2).

The differences between the calculated spectra and measured ones may be caused by several factors. Smaller amplitude is probably caused by the fact that the optical spectrum analyzer samples the spectrum in a sequence of wavelength intervals leading to some averaging of the acquired signal. The light source instability and a non-ideal measurement head positioning, as well as non-ideal nanocrystalline diamond sheet placement in the real laboratory conditions also have an impact on noted differences. However, the inconsistencies are small and the measured and calculated results remain in agreement.

## 4. Conclusions

The results show that we can tailor the properties of the Fabry–Perot cavities with different materials used for the mirror construction. Various refractive indices directly impact reflectivities of both boundaries, which changes the cavity finesse. In investigated cases, the silver mirror assures the highest finesse coefficient, while boron-doped diamond mirror the lowest. Tailoring of the cavity finesse is important in modeling the optoelectronic systems to better suit the requirements. Depending on the desired application, different values of finesse will increase the performance of the device, e.g., in an opto-electrochemical setup where optical parameters of the resonator can be tuned to match optical parameters of the investigated solution.

## Figures and Tables

**Table 1:** Parameters of the investigated cases: A—silver mirror; B—boron-doped diamond; C—nitrogen-doped diamond; D—nanocrystalline diamond sheet with silver mirror.

Parameter	A	B	C	D
Finesse coefficient	0.4891	0.3094	0.3653	4.4383
Minimal value	0.6716	0.7637	0.7324	0.2253

**Table 2:** Label convention of registered signals.

	A	B	C	D
Reflective surface	Silver	Boron-doped diamond	Nitrogen-doped diamond	Nanocrystalline diamond sheet and silver
Cavity length	100 µm	100 µm	150 µm	180 µm

## Data Availability

Data can be found at the open repository https://doi.org/10.34808/hv60-tf19 (accessed on 17 February 2022). or obtained directly from the corresponding authors.
